# Female Listeners’ Autonomic Responses to Dramatic Shifts Between Loud and Soft Music/Sound Passages: A Study of Heavy Metal Songs

**DOI:** 10.3389/fpsyg.2016.00182

**Published:** 2016-02-17

**Authors:** Tzu-Han Cheng, Chen-Gia Tsai

**Affiliations:** ^1^Department of Psychology, National Taiwan UniversityTaipei, Taiwan; ^2^Graduate Institute of Musicology, National Taiwan UniversityTaipei, Taiwan; ^3^Center for Neurobiology and Cognitive Science, National Taiwan UniversityTaipei, Taiwan

**Keywords:** arousal, heart rate, heavy metal music, relaxation, respiration rate

## Abstract

Although music and the emotion it conveys unfold over time, little is known about how listeners respond to shifts in musical emotions. A special technique in heavy metal music utilizes dramatic shifts between loud and soft passages. Loud passages are penetrated by distorted sounds conveying aggression, whereas soft passages are often characterized by a clean, calm singing voice and light accompaniment. The present study used heavy metal songs and soft sea sounds to examine how female listeners’ respiration rates and heart rates responded to the arousal changes associated with auditory stimuli. The high-frequency power of heart rate variability (HF-HRV) was used to assess cardiac parasympathetic activity. The results showed that the soft passages of heavy metal songs and soft sea sounds expressed lower arousal and induced significantly higher HF-HRVs than the loud passages of heavy metal songs. Listeners’ respiration rate was determined by the arousal level of the present music passage, whereas the heart rate was dependent on both the present and preceding passages. Compared with soft sea sounds, the loud music passage led to greater deceleration of the heart rate at the beginning of the following soft music passage. The sea sounds delayed the heart rate acceleration evoked by the following loud music passage. The data provide evidence that sound-induced parasympathetic activity affects listeners’ heart rate in response to the following music passage. These findings have potential implications for future research on the temporal dynamics of musical emotions.

## Introduction

Although music is a time-oriented structure that flows from emotion to emotion, the psychological effects of contrasting emotions in music remain poorly understood. A listener’s preference for contrasting musical emotions may relate to the preference for novelty. High predictability arising from repeated exposure to similar musical emotions may lead to habituation or desensitization (i.e., boredom) so that a novel stimulus is liked because it elicits an intensified emotional response (Schubert, 1996). A number of studies have shown that people prefer novel visual objects and places (Fantz, 1964; Berlyne, 1970; Olst, 1971; Bevins and Bardo, 1999). In the animal model, the reward system in the brain shows great sensitivity to novel stimuli. The mesolimbic dopamine reward pathway is activated when novel stimuli are approached (Bardo et al., 1996). A listener’s preference for novelty may explain why a majority of musical forms are based on melodic, rhythmic, tonal, timbral, dynamic, or textual contrasts between adjacent passages.

In addition to novelty, the effect of hedonic *contrast* caused by a shift of emotional character (e.g., from sadness to happiness) in music may lead to listeners’ greater appreciation. The hedonic-contrast effect refers to an effect whereby a test stimulus is judged in the opposite direction of a preceding (context) stimulus (Zellner et al., 2003). Parker et al. (2008) reported that musical pieces tend to be evaluated positively if they follow bad musical pieces (positive hedonic contrast), whereas musical pieces tend to be evaluated negatively if they follow good musical pieces (negative hedonic contrast). Schellenberg et al. (2012) showed that emotional contrasts between adjacent musical stimuli intensify listeners’ emotional responses, and the intensified emotional responses tend to increase listeners’ appreciation because music evoking strong emotions is preferred by listeners. Notably, hedonic-contrast effects have been particularly associated with responses to stimuli that are evaluated esthetically (Parducci, 1995; Dolese et al., 2005; Zellner et al., 2010).

In a musical piece, the conveyed emotion can move along two dimensions commonly used to describe emotion: valence and arousal (Russell, 1980). Contrasts of emotional valence can be observed in masterpieces of Western classical music such as Beethoven’s Fifth Symphony and Tchaikovsky’s Fifth Symphony, in which the archetype of the strife to victory plot is implemented by the transformation of a minor mode to a major mode (Kraus, 1991). On the other hand, the verse-chorus form in popular music benefits from the shifts between the low-to-moderate arousal level associated with the verse and the high arousal level associated with the chorus. Recently, Tsai et al. (2014) provided supportive evidence that the cathartic effect of popular sad songs may stem from the reinterpretation of the musical materials within the second verse-to-chorus progression and the third chorus that contain dynamic changes. It appears that the adaptive significance and functions of negatively valenced music may relate to a listener’s intertemporal integration and esthetic appraisal of musical emotions.

Compared with sad music, the benefits of exposure to angry music have been relatively under-studied. A representative genre of angry music may be heavy metal music, which often expresses extreme fury and aggression using highly distorted sounds of electric guitars, emphatic rhythms, dense bass and drum sounds, growling vocals, and an overall intense sound level. It has been suggested that exposure to heavy metal music may increase the risk of depression, delinquency, and suicide (Stack et al., 1994; Gowensmith and Bloom, 1997; Selfhout et al., 2008; Shafron and Karno, 2013). A contrary view suggests that heavy metal music can reduce listeners’ negative emotions by distracting the listener from a negative mood or discharging negative feelings (Arnett, 1995; Schwartz and Fouts, 2003). A recent study demonstrated that extreme metal music appeared to match listeners’ physiological arousal when the listeners were angry, thereby increasing positive emotions (Sharman and Dingle, 2015). To date, empirical evidence remains scant as to whether and how heavy metal music leads to positive effects in listeners.

A special technique in heavy metal music uses dramatic changes between loud and soft dynamics. Loud passages are penetrated by a *death growl*, which is characterized by a harsh timbre and noisy background. By contrast, soft passages are often characterized by a clean, calm singing voice and light, lyrical accompaniment. Heavy metal music excerpts with dramatic shifts between loud and soft passages provide a good model to test how listeners respond to the temporal dynamics of musical emotion. A shift from a loud passage to a soft passage in a heavy metal song is not only associated with a decrease in arousal level but is also associated with a decrease in musical tension. Given that a greater musical tension often expresses a high arousal level, loudness is a key determinant of perceived tension (Granot and Eitan, 2011). Moreover, loud passages in heavy metal music are characterized by growl-like timbres of the singing voice and the electric guitars that are likely perceived as aggressive expressions (Tsai et al., 2010). The growl-like timbres in loud heavy metal music can create (1) perceptual dissonance via a high value of acoustic roughness (Daniel and Weber, 1997) and (2) cognitive-emotional instability because of a strong motivational drive to escape from the aggressive intention expressed by the music. Therefore, when the aggressive intention is terminated by the lyrical, calming sounds of the soft passage, the listener tends to feel stable and relaxed.

The goal of this study is to examine female listeners’ autonomic responses to passages of contrasting arousal and tension. While previous studies have demonstrated how listeners respond to the temporal dynamics of musical emotion (Schubert, 2004; Bachorik et al., 2009; Egermann et al., 2009; Coutinho and Cangelosi, 2011; Tsai et al., 2014; Tsai and Chen, 2015), the dramatic shifts between a loud, aggressive passage and a soft, relieving passage may shed new light on how the time-varying music-expressed emotion affects listeners. We only recruited female participants because loud heavy metal music seems to consistently evoke intense negative feelings in female listeners (Nater et al., 2006). The experiment comprised a within-participants repeated-measures design with the factors of (1) the arousal level expressed by the first passage and (2) the arousal level expressed by the second passage. Respiration rate and heart rate were used to assess listeners’ autonomic arousal levels.

We performed a heart rate variability (HRV) analysis and used its high-frequency (0.15–0.40 Hz) power to quantify listeners’ parasympathetic activity (Task Force, 1996; Pumprla et al., 2002). It has been amply documented that the high-frequency power of heart rate variability (HF-HRV) decreases in stress states (Mazurak et al., 2013; Chalmers et al., 2014; Kuehl et al., 2015; Visnovcova et al., 2015). High levels of HF-HRV are linked to better performance on executive function (Mann et al., 2015), which mediated the correlation between HF-HRV and cheerfulness/calmness (Geisler et al., 2010). A few studies have demonstrated that relaxing music increase listeners’ HF-HRV (Okada et al., 2009; Peng et al., 2009; Chuang et al., 2010, 2011; da Silva et al., 2014). We predicted that participants’ HF-HRVs will be higher during exposure to the soft passages of heavy metal songs and soft sea sounds compared with the loud passages of heavy metal songs.

According to the effect of positive hedonic contrast, we hypothesized that the arousing loud passage may enhance the relaxing effect of the ensuing soft passage in heavy metal songs. The loud-to-soft progressions would decrease the heart and respiration rates to levels lower than the rates achieved after the progression from the soft sea sounds to the soft music passage. The first hypothesis investigated is as follows:

Hypothesis 1 (hedonic contrast): Compared with the soft sea sounds, the loud music passage enhances the relaxing effect of the ensuing soft music passage, as revealed in the over-deceleration of the heart rate and respiration rate at the beginning of the soft music passage (the second passage).

In addition to loud-to-soft transitions, the present study also included soft-to-loud progressions. We proposed two opposing hypotheses regarding this type of arousal change. One hypothesis, based on the negative hedonic contrast, claims that the soft, relaxing passage may enhance the arousing effect of the following loud, aggressive passage. The other hypothesis claims that the listener’s relaxation caused by the soft passage may reduce and/or delay the arousing effect of the ensuing loud passage. A similar notion in clinical psychology, emotional inertia, refers to the degree to which emotional states are resistant to change (Kuppens et al., 2010). This notion was originally related to depressive symptoms and prospectively predicts the onset of depression as an early marker (Kuppens et al., 2010, 2012; Koval et al., 2012). In the present study, great emotional inertia refers to the fact that the listener maintains the high level of parasympathetic activity after the soft-to-loud transition. The two opposing hypotheses regarding the listener’s emotional responses to the soft-to-loud transitions are as follows:

Hypothesis 2A (hedonic contrast): Compared with the loud music passage, the soft sea sound enhances the arousing effect of the ensuing loud music passage, as revealed in the over-acceleration of the heart rate and respiration rate at the beginning of the loud music passage (the second passage).

Hypothesis 2B (emotional inertia): Compared with the loud music passage, the soft sea sounds reduce the arousing effect of the ensuing loud music passage, as revealed in the delayed acceleration of the heart rate and respiration rate at the beginning of the loud music passage (the second passage).

## Materials and Methods

### Participants

Thirty-three female participants, aged between 20 and 27 years, were recruited via online advertisements. All participants reported having no neurological, mental, or hearing problems. None of the participants were professional musicians. Participants were provided with a written and verbal explanation of the entire experimental procedure and gave informed written consent. The data of one participant were not included in data analyses because she fell asleep during the experiment. The protocol of the study was approved by the Research Ethics Committee of the National Taiwan University.

### Stimuli

There are two categories of stimuli: a music-music type and a seawave-music type. Each type comprised six excerpts. Stimuli of the music–music type comprised six excerpts of 60 s duration (with a 1 s fade-in and a 1 s fade-out) extracted from six heavy metal songs. Three stimuli of this type comprised a 30 s loud passage and then a 30 s soft passage; the other three stimuli were loud throughout. Stimuli of the seawave-music type were generated by substituting the first half (30 s) of the stimuli of the music-music type with soft sea sounds, which presumably relaxes listeners and relieves their pain (Nilsson et al., 2001; Good et al., 2002; Lin et al., 2011). In all, we created four manipulated conditions: loud music plus loud music (Loud + Loud), loud music plus soft music (Loud + Soft), soft sea sounds plus loud music (Soft + Loud), and soft sea sounds plus soft music (Soft + Soft). There was no fade-out or fade-in at the transition between the first and second passages. We calculated the values of root mean square (RMS) energy of the soft passages and loud passages using the MIR toolbox for Matlab (Mathworks, Inc.).

### Procedure

Participants were seated in an armchair at ease in a sound-attenuated room with a comfortable temperature (Celsius temperature 25–27°C). Participants provided informed written consent and were given verbal explanation of the entire experimental procedure. Before the physiological measures, the participants were requested to rate the arousal expressed by music and their familiarity with two 5 s excerpts of each stimulus (20–25 s and 50–55 s) on a 7-point scale ranging from “not at all” (1) to “extreme” (7). The participants also rated the sound-expressed arousal level and their familiarity with soft sea sounds. This portion of the experiment took approximately 5 min. Participants were then instructed to close their eyes and to focus on the auditory stimuli. Twelve stimuli were presented in random order through two speakers in front of the participants. The presentation of each stimulus was preceded by 4 s of silence, a warning tone (440 Hz, 100 ms), and another 1 s of silence. We assumed that this inter-trial interval was enough for the listeners’ physiological-emotional signals to return to levels close to the baseline, and the subsequent sound/music was likely to rapidly influence listeners’ emotion. Participants spent approximately 20 min. completing this portion of the experiment.

### Data Acquisition

Three physiological measures were obtained using a Biopac MP35 system (Biopac Systems Inc., USA), including respiration rate, heart rate, and finger temperature. Respiration was assessed by a belt placed on the chest that contained a piezoelectric sensor responsive to changes in thoracic circumference. Electrocardiogram was measured by electrodes placed under the right collarbone, left rib and wrist of the non-dominant hand. Finger temperature was recorded by placing a temperature probe on the volar surfaces of the medial phalanges of the ring finger on the non-dominant hand. Because of the abnormally high values of finger temperatures, we will not include finger temperature data in the remainder of this paper.

Respiration signals were downsampled to 10 Hz, and a moving time window of 10 s in duration was used to extract the time-averaged respiration rate. By applying a Fast Fourier Transform (FFT), the respiration signals within this time window were transformed to the frequency domain, and the power spectrum was obtained. The time-averaged respiration rate of the middle point of this time window was the frequency of the spectral peak.

The instant heart rate was derived from the R–R intervals in the electrocardiogram signal. The HF-HRV was evaluated by applying a FFT with 30-s Kaiser window to the instant heart rate data within the first and second halves of each stimulus, and by integrating the power spectra over 0.15–0.40 Hz. The HF-HRV was logarithmically transformed to normalize the distribution (Kobayashi et al., 2012).

A moving 10-s time window was used to extract the time-averaged heart rate. The time-averaged heart rate of the middle point of this time window was the median of the instant heart rates within this window. This extent of smoothing was sufficient to suppress signal noise.

### Statistical Analysis

All tests conducted were two-tailed with an alpha value of 0.05. All excerpts were divided into the loud group and the soft group according to perceived loudness. For self-report inventory, the arousal scores of these two groups were subjected to a *t*-test for differences between the loud and soft passages. For the sound-induced parasympathetic activity, the natural logarithmic transformed values of the HF-HRV of loud group and the soft group were subjected to a *t*-test. Differences of HF-HRV within the loud group and soft group were analyzed by one-way ANOVAs, respectively.

Analyses of the respiration rate and heart rate were based on the arousal levels expressed by the stimuli. We inferred that the soft sea sounds and the soft music passages expressed low arousal, while the loud music passages expressed high arousal. Two-way 2 (expressed arousal level of the first 30 s passage: high, low) × 2 (expressed arousal level of the second 30 s passage: high, low) repeated-measures analyses of variance (ANOVAs) were conducted at three given time points (*t*1 = 35 s, *t*2 = 45 s, and *t*3 = 55 s). These analyses examined whether the participant’s physiological-emotional responses at different time points during the second passage were influenced by the arousal levels expressed by the first passage and second passage.

## Results

### Stimulus Loudness and Self-Reported Ratings

The stimuli and descriptive analysis of self-reported ratings are displayed in **Table 1.** The loud music group includes excerpts 1, 3, 5, and 7–12; the soft group includes the musical excerpts 2, 4, 6, and the soft sea sounds (excerpt 13). The mean values of RMS energy, arousal, and familiarity were presented in **Table 1.** The values of RMS energy of the loud passages are higher than those of the soft passages. The participants assigned high and low arousal scores for the loud and soft groups, respectively. A *t*-test shows a significant difference in expressed arousal between the two groups (*t*[31] = 3.06, *p* < 0.001, 95% CI[0.237,2.579]). The loud passages were rated as conveying higher arousal than the soft passages. Moreover, the participants generally assigned low familiarity scores to these heavy metal songs.

**Table 1:** The auditory stimuli and the results of root mean square (RMS) energy, self-reported ratings of sound-conveyed arousal and familiarity on a 7-point scale.

Song title / Stimulus	Band	Time span	RMS energy	Arousal (mean ±*SD*)	Familiarity (mean ±*SD*)
01 Decline (1st, loud)	Agathodaimon	4:34–5:03	0.2505	5.50 ± 1.30	2.53 ± 1.54
02 Decline (2nd, soft)	Agathodaimon	5:04–5:33	0.1156	2.84 ± 1.27	2.69 ± 1.53
03 Ghost of Perdition (1st, loud)	Opeth	2:05–2:34	0.2852	5.10 ± 1.16	2.56 ± 1.48
04 Ghost of Perdition (2nd, soft)	Opeth	2:35–3:04	0.1189	3.16 ± 1.55	3.00 ± 1.76
05 Revenge of the Dadaist (1st, loud)	The Agonist	3:10–3:39	0.2231	5.20 ± 1.28	2.78 ± 1.70
06 Revenge of the Dadaist (2nd, soft)	The Agonist	3:40–4:09	0.1120	2.30 ± 1.30	3.84 ± 1.78
07 Ghost Walking (1st, loud)	Lamb Of God	0:36–1:05	0.3043	5.91 ± 1.30	2.84 ± 1.90
08 Ghost Walking (2nd, loud)	Lamb Of God	1:06–1:35	0.2979	6.09 ± 1.20	3.13 ± 2.03
09 Ruin (1st, loud)	Lamb Of God	0:45–1:14	0.2518	5.59 ± 1.13	3.06 ± 1.97
10 Ruin (2nd, loud)	Lamb Of God	1:15–1:44	0.2317	5.91 ± 1.06	2.97 ± 1.86
11 Laid to Rest (1st, loud)	Lamb Of God	1:11–1:40	0.2938	5.60 ± 1.07	2.53 ± 1.60
12 Laid to Rest (2nd, loud)	Lamb Of God	1:41–2:10	0.2997	5.80 ± 1.11	2.75 ± 2.00
13 Sea Sound (soft)	YouTube (https://www.youtube.com/watch?v=wGKIjh9skYw)	0:00–0:29	0.0586	2.03 ± 1.38	4.84 ± 1.92

### Physiological-Emotional Signals

**FIGURE 1:**
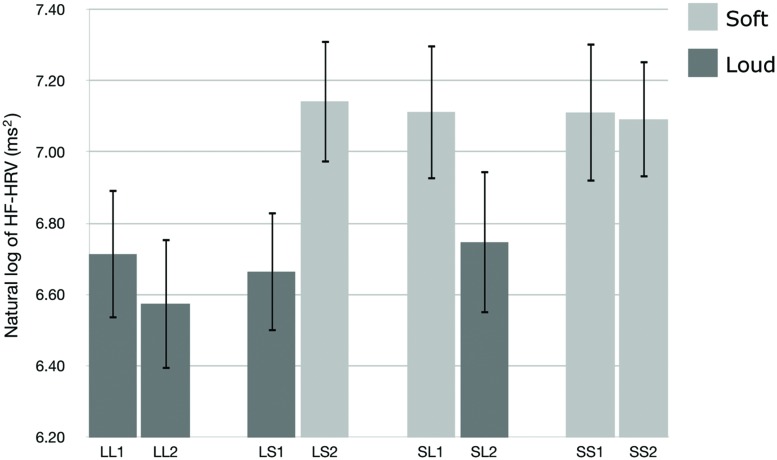
**The natural logarithm of high-frequency heart rate variability (HF-HRV) for the first and second halves of the Loud + Loud (LL), Loud + Soft (LS), Soft + Loud (SL), and Soft + Soft (SS) stimuli.** Error bars indicate standard error of mean.

We used HF-HRV to assess listeners’ cardiac parasympathetic activity during exposure to the auditory stimuli. **Figure 1** presents the natural logarithm of HF-HRV for the first and second passages of the Loud + Loud, Loud + Soft, Soft + Loud, and Soft + Soft stimuli. A *t*-test shows a significant difference in the natural logarithm of HF-HRV between the loud group and the soft group (*t*[31] = -7.614, *p* < 0.001, 95% CI[0.557,0.322)]). The soft passages induced higher HF-HRV than the loud passages. One-way ANOVAs showed that there was no significant difference among the loud passages [*F*(3,124) = 0.238, *p* = 0.870, η^2^ = 0.006], and there was no significant difference among the soft passages [*F*(3,124) = 0.013, *p* = 0.998, η^2^ = 0.000].

**FIGURE 2:**
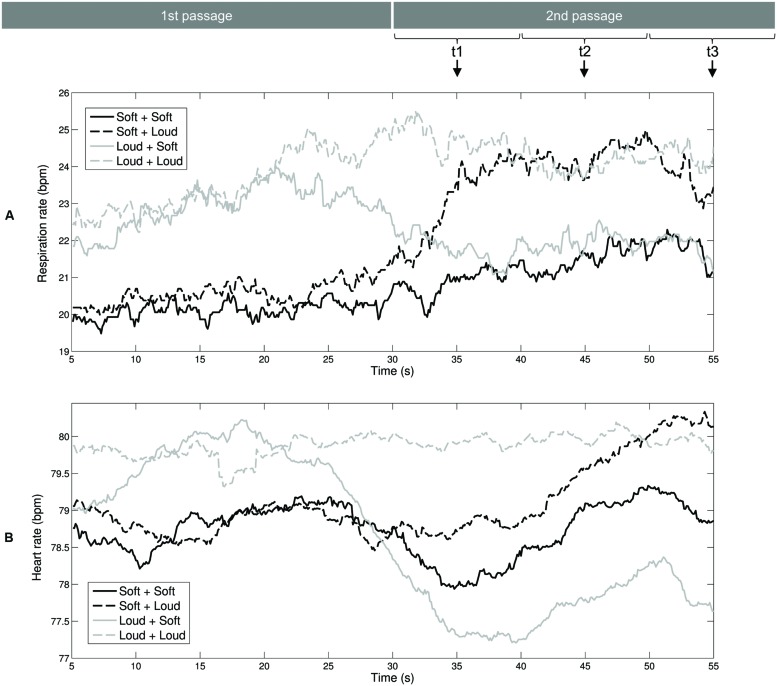
**Mean curves of the physiological-emotional signals during exposure to the auditory stimuli.** **(A)** Respiration rate. **(B)** Heart rate. These two rates were averaged within a 10-s window. Therefore, the values at the three time points (*t*1, *t*2, and *t*3) selected for ANOVAs correspond to the time windows of 30–40, 40, and 50–60 s, respectively.

We used respiration rate and heart rate to assess listeners’ autonomic arousal levels during exposure to the auditory stimuli. **Figure 2A** shows the mean curves of the respiration rate. For the Loud + Soft and Soft + Loud stimuli, the changes in arousal level led to shifts in the respiration rate at approximately 33 s. The two black curves of the music stimuli starting with soft sea sounds are approximately coincident within 5–20 s, and the two gray curves of the music stimuli starting with loud music are approximately coincident within the same time range. Identical patterns can be observed with the time range of 40–55 s. **Figure 2B** shows the mean curves of the heart rate. The shift from the high-arousal passage to the low-arousal passage (stimulus Loud + Soft) led to a decrease in the heart rate at 30 s (the gray solid curve). The shift from the soft sea sounds to loud music (stimulus Soft + Loud) led to a delayed increase of the heart rate at approximately 45 s (the black dashed curve).

**FIGURE 3:**
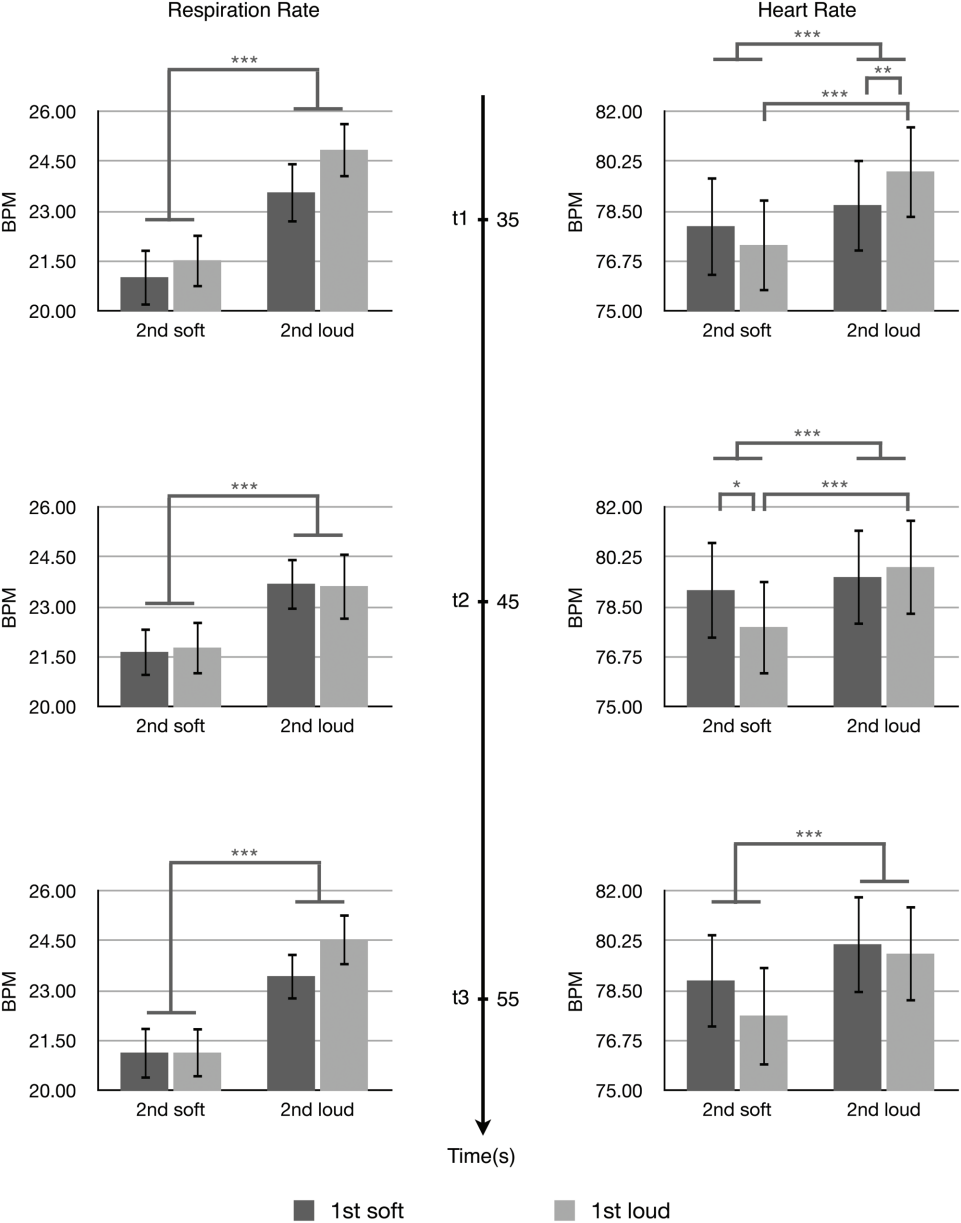
**The respiration rate and heart rate at *t*1, *t*2, and *t*3.** Error bars indicate standard error of mean. ^∗^*p* < 0.05, ^∗∗^*p* < 0.01, ^∗∗∗^*p* < 0.001.

The effects of the arousal levels expressed by the first and second passages on listeners’ physiological-emotional responses to the second passages were analyzed by two-way ANOVAs. **Figure 3** shows the results of respiration rate and heart rate at three time points. For the respiration rate, there was a significant main effect of the expressed arousal level of the second passages at all three time points; *t*1, *F*(1,31) = 27.392, *p* < 0.001, η^2^ = 0.469; *t*2, *F*(1,31) = 14.948, *p* = 0.001, η^2^ = 0.325; *t*3, *F*(1,31) = 36.262, *p* < 0.001, η^2^ = 0.539. The second passages conveying higher arousal induced higher respiration rates compared with the second passages conveying lower arousal. There was a significant main effect of the arousal level conveyed by the first passages at *t*1, *F*(1,31) = 5.231, *p* = 0.029, η^2^ = 0.144. The first passages conveying higher arousal induced higher respiration rates at *t*1 (the beginning of the second passage) compared with the first passages conveying low arousal. No statistical significance was observed for the interaction between the expressed arousal levels of the first and second passages to the respiration rate.

For the heart rate, two-way ANOVAs showed a significant main effect of the arousal level conveyed by the second passages at all three time points; *t*1, *F*(1,31) = 25.343, *p* < 0.001, η^2^ = 0.450; *t*2, *F*(1,31) = 16.741, *p* = 0.001, η^2^ = 0.325; *t*3, *F*(1,31) = 23.848, *p* < 0.001, η^2^ = 0.539. The second passages conveying higher arousal induced higher heart rates compared with the second passages conveying lower arousal.

There were significant interactions between the arousal levels conveyed by the first and second passages to the heart rate at *t*1, *F*(1,31) = 7.477, *p* = 0.010, η^2^ = 0.194 and *t*2, *F*(1,31) = 7.647, *p* = 0.009, η^2^ = 0.198. These interactions were characterized *post hoc* with Bonferroni corrected *t*-tests. At *t*1, the stimulus Loud + Loud led to significantly higher heart rates compared with the stimulus Soft + Loud (*t*[31] = -2.845, *p* = 0.008, 95% CI[-2.03066,-0.33479]), and the stimulus Loud + Loud induced significantly higher heart rates than the stimulus Loud + Soft (*t*[31] = 5.682, *p* < 0.001, 95% CI[3.50535,5.682]). At *t*2, the stimulus Soft + Soft caused a significant increase in the heart rate compared with the stimulus Loud + Soft (*t*[31] = 2.286, *p* = 0.029, 95% CI[0.13984,2.45409]), and the stimulus Loud + Loud induced a significant increase in the heart rate compared with the stimulus Loud + Soft (*t*[31] = 4.836, *p* < 0.001, 95% CI[1.21329,2.98285]).

## Discussion

Listeners’ experiences of felt emotion during music listening unfold over time, and this experience relies on the integrative processing of the present passage and the preceding passages. In this study, we investigated the relation between female listeners’ autonomic responses and the temporal dynamics of arousal/tension conveyed by music/sound. There were four manipulated conditions: Loud + Loud, Loud + Soft, Soft + Loud, and Soft + Soft. In line with a few previous studies (Okada et al., 2009; Peng et al., 2009; Chuang et al., 2010, 2011; da Silva et al., 2014), the result of HF-HRV showed that the soft, relaxing passages induced stronger parasympathetic activity compared with the loud passages. We observed that the delayed changes of the respiration rate were determined by the arousal level expressed by the present passage, whereas the heart rate changes were determined by the arousal levels expressed by the present passage and the preceding passage. The loud music passage (inducing lower parasympathetic activity) effectively enhanced the relaxing effect of the ensuing soft music passage, as revealed in the over-deceleration of the heart rate at the beginning of the soft music passage. Conversely, the soft sea sounds (inducing higher parasympathetic activity) effectively delayed the arousing effect of the ensuing loud music passage, as revealed in the delayed heart rate acceleration.

### Respiration Rate

The finding that the respiration rate was dependent on the arousal level expressed by the present passage is consistent with previous studies demonstrating relations between respiration and emotional arousal (Boiten, 1998; Van Diest et al., 2001). In the field of music psychology, Krumhansl (1997) reported increases in respiration rate during exposure to clips portraying high-arousal emotions such as happiness and fear compared with baseline. Given that heavy metal music may lead to intense negative feelings in female listeners (Nater et al., 2006), the increased respiration rate observed in our study was likely to reflect fear or anxiety evoked by the loud passages of heavy metal songs.

The respiration rate showed a short delay (<10 s) in response to arousal changes, as revealed by the main effect of the arousal level of the first passage at *t*1. Hypothesis 2A was not supported and Hypothesis 2B was partially supported with regard to the respiration rate. For *t*2 and *t*3, the respiration rate was independent of the arousal level of the preceding passage. Hypothesis 1 was not supported with regard to respiration rate. These results suggest that although the respiration rate showed a short delay in response to arousal changes, respiration appeared unaffected by the higher-level processing of the intertemporal integration of music.

### Heart Rate

In addition to the respiration rate, heart rate is also an index of physiological arousal. High-arousal music is likely to induce a higher level of sympathetic activity, which leads to increased heart rate and respiration rate. Prior studies have shown that listeners’ heart rates are significantly higher during exposure to high-arousal music, whereas heart rates are significantly lower during exposure to sedative music (Knight and Rickard, 2001; Etzel et al., 2006; Grewe et al., 2009; Salimpoor et al., 2009; Koelsch and Jäncke, 2015; Tsai and Chen, 2015). A novel finding of our study was that listeners’ heart rates were influenced by the arousal level expressed by the preceding passage, with the loud-to-soft and soft-to-loud progressions manifesting different effects.

The first effect was analogized as hedonic contrast, an effect whereby a test stimulus is judged in the opposite direction of a preceding (context) stimulus (Zellner et al., 2003). Although the measure of HF-HRV has a low temporal resolution so that it did not indicate the transient relaxation after the loud-to-soft progressions, we observed that the heart rate during the initial 20 s of the second passage was significantly lower for the stimulus Loud + Soft than for the stimulus Soft + Soft (**Figure 2B**), and this effect vanished thereafter. Hypothesis 1 was supported with regard to the heart rate, and we observed that the over-deceleration of the heart rate lasted 20 s. In the future, when a better understanding of the psychological/physiological mechanisms underlying this relaxing effect has developed, such music excerpts could be used for therapeutic purposes.

The second effect of the changing arousal associated with auditory stimuli was analogized as emotional inertia; the listener maintains the high level of parasympathetic activity after the soft-to-loud transition. The effect of emotional inertia was shown by the significantly lower heart rate in response to the stimulus Soft + Loud compared with the stimulus Loud + Loud during the initial 20 s of the second passage (30–50 s in **Figure 2B**), and this effect vanished thereafter. Hypothesis 2B was supported, and Hypothesis 2A was not supported with regard to heart rate. Physiologically, the delayed heart rate acceleration may be related to the fact that heart rate acceleration is caused by sympathetic nervous system activity through the release of norepinephrine (Task Force, 1996). However, it should be noted that the respiration rate acceleration showed a shorter delay than the heart rate acceleration in response to soft-to-loud transitions. Compared with the respiration rate, the heart rate may be mediated by higher-level neural mechanisms.

The contextual imagination associated with the sea sounds is one of the possible explanations for the delayed heart rate acceleration. Previous studies have demonstrated that the heart rate is higher during high-arousal than during low-arousal situational imagery (Acosta and Vila, 1990; Witvliet and Vrana, 1995). Although listening to music generated some imagery, environmental noise was more likely to stimulate mental images (Gomez and Danuser, 2004). Exposure to the sea wave noise with calls of sea gulls may automatically evoke images of the relaxing beach and sunshine, providing cues for spatial orientation. Orientation is an important index in neuropsychological assessment and describes a person’s awareness of himself or herself in relation to the place around them (Zillmer et al., 2008). Listening to the soft sea sounds may orient the participants to a relaxing beach. Therefore, participants may spend 20 s re-orienting to a high-arousal, high-tension state.

### Limitations and Future Directions

There were several limitations to the present study. First, the experiment was conducted in a laboratory under controlled conditions, not a natural setting such as at a concert. Second, we used ready-made songs and soft sea sounds as stimuli. Although it is generally accepted that soft sea sounds are relaxing, the combinations of soft sea sounds and excerpts of heavy metal music is uncommon and of low ecological validity. Third, we did not record physiological data in a baseline condition without any stimulation. Future investigations should compare the baseline data with those for loud-to-soft progressions and soft-to-soft progressions. Fourth, we did not consider several factors of individual differences such as musical preference/experience, physiological condition, and personality traits, which may affect listeners’ physiological-emotional responses to music. For example, Masaoka and Homma (1997) reported the effect of personality traits on the patterns of breathing during mental stress and physical load, and levels of individual anxiety also affect respiration rate and expiratory time (Masaoka and Homma, 1999, 2001). Vuoskoski and Eerola (2011) observed that Big Five scores can predict a dissimilar perception of music-expressed emotion. Unfortunately, we did not collect any data regarding participants’ personality traits.

Results of the current study suggest productive avenues for future research. This study only recruited female participants. Thus, it would be interesting to examine whether the same pattern of responses could be observed in male participants. Several participants expressed that they simultaneously experienced positive and negative emotions during exposure to the loud passages of our stimuli. Future investigations should use structured questionnaires and semi-structured interviews to collect information about the esthetic emotions evoked by various passages and transitions in heavy metal music. Of particular interest to the relaxed feeling evoked by the loud-to-soft transitions in heavy metal songs is the use of neuroimaging technologies for delineating the neural substrates of high-level musical emotions. Trost et al. (2012) showed activation in the medial orbitofrontal cortex and parahippocampal gyrus when experiencing low-arousal esthetic emotions to music, such as wonder and transcendence. We speculated that the mysterious, sacred atmosphere associated with the soft passages in heavy metal music may evoke transcendent emotions in listeners.

## Conclusion

To explore listeners’ intertemporal integration of musical emotions, this study systematically manipulated the arousal/tension levels conveyed by the first passage and the second passage of the stimuli. When the emotion conveyed by heavy metal music moved from aggression to peace, the heart rate was significantly lower compared with the same peaceful music passage preceded by relaxing sea sounds. This effect highlights the role of hedonic contrast in music appreciation. In line with recent studies on the progression of reward-anticipation to reward-gain in music (Salimpoor et al., 2011; Tsai et al., 2014; Li et al., 2015), we have shown how the temporal dynamics of musical arousal/tension affect listener’s physiological-emotional responses Although this study sheds new light on the positive psychological effects of heavy metal music, more investigations are required to compare the time-varying nature of musical emotions in different genres and forms.

## Author Contributions

CT and TC designed this study and wrote the draft of this manuscript. TC collected the data from participants and performed the statistical calculations.

## Conflict of Interest Statement

The authors declare that the research was conducted in the absence of any commercial or financial relationships that could be construed as a potential conflict of interest.
